# Editors' Reply

**DOI:** 10.1371/journal.pmed.0020152

**Published:** 2005-05-31

**Authors:** Virginia Barbour, Barbara Cohen, Gavin Yamey

We agree with Doug Altman that correspondence is an essential part of medical publishing—acting as post-publication peer review. Hence, we encourage submission of letters in response to articles we publish, preferably as e-letters via our Web site (www.plosmedicine.org) rather than via our manuscript submission site. The four-week limit we originally suggested in our author guidelines was to encourage timely correspondence; however, we accept that there will be occasions when it will be appropriate for letters to be published later than that, and we will accept such submissions. The word limit is more debatable. We think that 750 words is a reasonable limit; any more, and letters are likely to stray off the topic.

